# Microbial Iron Mats at the Mid-Atlantic Ridge and Evidence that Zetaproteobacteria May Be Restricted to Iron-Oxidizing Marine Systems

**DOI:** 10.1371/journal.pone.0119284

**Published:** 2015-03-11

**Authors:** Jarrod J. Scott, John A. Breier, George W. Luther, David Emerson

**Affiliations:** 1 Bigelow Laboratory for Ocean Sciences, East Boothbay, Maine, United States of America; 2 Woods Hole Oceanographic Institute, Woods Hole, Massachusetts, United States of America; 3 College of Earth, Ocean, and Environment, University of Delaware, Lewes, Delaware, United States of America; Universite Pierre et Marie Curie, FRANCE

## Abstract

Chemolithoautotrophic iron-oxidizing bacteria play an essential role in the global iron cycle. Thus far, the majority of marine iron-oxidizing bacteria have been identified as Zetaproteobacteria, a novel class within the phylum Proteobacteria. Marine iron-oxidizing microbial communities have been found associated with volcanically active seamounts, crustal spreading centers, and coastal waters. However, little is known about the presence and diversity of iron-oxidizing communities at hydrothermal systems along the slow crustal spreading center of the Mid-Atlantic Ridge. From October to November 2012, samples were collected from rust-colored mats at three well-known hydrothermal vent systems on the Mid-Atlantic Ridge (Rainbow, Trans-Atlantic Geotraverse, and Snake Pit) using the ROV *Jason II*. The goal of these efforts was to determine if iron-oxidizing Zetaproteobacteria were present at sites proximal to black smoker vent fields. Small, diffuse flow venting areas with high iron(II) concentrations and rust-colored microbial mats were observed at all three sites proximal to black smoker chimneys. A novel, syringe-based precision sampler was used to collect discrete microbial iron mat samples at the three sites. The presence of Zetaproteobacteria was confirmed using a combination of 16S rRNA pyrosequencing and single-cell sorting, while light micros-copy revealed a variety of iron-oxyhydroxide structures, indicating that active iron-oxidizing communities exist along the Mid-Atlantic Ridge. Sequencing analysis suggests that these iron mats contain cosmopolitan representatives of Zetaproteobacteria, but also exhibit diversity that may be uncommon at other iron-rich marine sites studied to date. A meta-analysis of publically available data encompassing a variety of aquatic habitats indicates that Zetaproteobacteria are rare if an iron source is not readily available. This work adds to the growing understanding of Zetaproteobacteria ecology and suggests that this organism is likely locally restricted to iron-rich marine environments but may exhibit wide-scale geographic distribution, further underscoring the importance of Zetaproteobacteria in global iron cycling.

## Introduction

Understanding the distribution of microorganisms and specific microbial assemblages continues to be a core pursuit of microbial ecology. Resources are thought to be primary factors that influence microbial distribution and principal drivers of community diversity [1]. In many environments (e.g., the deep ocean), resource islands—localized concentrations of differentially abundant resources [2]—may have a considerable impact on biodiversity and community assembly [3–5]. This is particularly clear for chemolithoautotrophic systems in which specialized microorganisms utilize inorganic energy sources that in turn serve as the basis for community-level dynamics (reviewed in [6]). This habitat restriction, coupled with the patchy distribution of many chemolithoautotrophic systems [7], makes these important ecological models for understanding biogeography, recruitment processes, gene flow, and community assembly [8,9]. In addition, these systems may have important consequences for the establishment of novel food webs [10–12], the development of synergistic associations [13,14], and the rise of evolutionary specialization [15,16].

In marine environments, chemolithoautotrophic metabolism is prominent at hydrothermal vent ecosystems [17,18] where superheated fluids from the deep subsurface interact with cold seawater [19]. Once discharged, these fluids cool rapidly and a variety of minerals precipitate out [20], often forming distinct chimney-like structures [21,22]. Since their discovery in the late 1970s [20], numerous studies have demonstrated that hydrothermal systems harbor unusual assemblages of both micro- and macro-organisms (reviewed in [23]), driven in large part by the unique physiochemical properties of the vent fluid. Depending on the hydrothermal system, these fluids can exhibit different chemical composition, though fluids generally share many common features. In particular, these fluids are highly reduced and acidic, as well as enriched in a variety elements, including sulfur and iron [24].

Iron can be a plentiful energy source due to its global abundance [25] and role as an electron donor for chemolithoautotrophic growth via oxidation of iron(II) to iron(III) [26,27], despite that the free energy available from iron oxidation is low [28]. The kinetics of abiotic iron(II) oxidation at circumneutral pH restricts oxygen-dependent, neutrophilic iron-oxidizing bacteria (FeOB) to microaerobic environments [29]. Nonetheless, FeOB and their associated communities are prevalent wherever anoxic ferrous-rich subsurface waters mingle with oxygenated surface waters (reviewed in [26]) (see also [30–34]). Because the ambient temperature is 2°C, abiotic oxidation rates decrease by more than a factor of four (compared to room temperature), which favors biotic iron-oxidation. In addition to being important primary producers in these ecosystems, FeOB also generate centimeters-thick microbial mats (iron mats) as a byproduct of iron-oxyhydroxide precipitation [35]. These mats can influence water flow and local chemical composition in addition to providing large surface areas that can be colonized by other bacteria [36]. Iron-oxyhydroxide encrusted structures are easily recognized by light microscopy [37]. This physiological adaptation also has the added benefit of acting as a biomarker for biotic iron-oxidation [37].

The most abundant assemblages of FeOB in marine environments have been associated with volcanically active seamounts [31]. Initial identification of these FeOB was based on the morphological similarity of biomineral structures recovered from seamounts in the Pacific [38] to the iron-oxyhydroxide encrusted stalks from the well-known freshwater FeOB *Gallionella ferruginea* [39,40]. However, molecular and cultivation-based studies revealed that the Zetaproteobacteria, a novel class of Proteobacteria, are in fact the predominant FeOB in marine systems [41]. The first isolate from the marine environment, *Mariprofundus ferrooxydans*, is an obligate FeOB with a bean-shaped cell that produces a helical stalk [41]—very reminiscent of *Gallionella ferruginea*—however, genomic analysis demonstrated that these two organisms share few genes in common [27,42]. Further work has shown that FeOB at vents are part of structured microbial mat communities that appear to be shaped by a confluence of physicochemical factors [31,32]. It is apparent that a steady flux of iron(II) from anoxic vent fluids intruding into oxygenated seawater controls the overall presence of iron-oxidizing communities; however, we do not understand the specifics of what drives community structure, community diversity, nor variation in FeOB populations at different sites. Though beyond the scope of this study, these issues remain central to better understanding the evolutionary ecology of FeOB.

Although FeOB have been found at a variety of seamounts and subduction centers, neither free-living Zetaproteobacteria nor iron mats have been identified at hydrothermal systems along the Mid-Atlantic Ridge (MAR), a slow-spreading ridge system running from 50°N to 50°S. In fact, based on a literature survey it was difficult to find evidence for the existence of iron-oxidizing communities at any crustal spreading zone. The MAR is known for a series of high-temperature (250–400°C) hydrothermal vent fields marked by prominent black smoker chimneys that support diverse biological communities. The goal of this study was to determine if microbial iron mats existed associated with diffuse flow venting (less than 100°C) proximal to black smokers at three well-studied hydrothermal sites along the MAR. Our working hypothesis was that iron-based ecosystems are common, though overlooked at black smoker sites, and that FeOB diversity at these sites would resemble those found in the Pacific, but also contain diversity unique to the MAR. Here we use a combination of pyrosequencing, light microscopy, and single-cell sorting to demonstrate the presence of iron-oxidizing Zetaproteobacteria at diffuse flow vent sites along the MAR as well as gain a better understanding of general bacterial diversity in these unusual and under-studied systems.

## Materials and Methods

### Study overview, site description and sampling

**Fig 1:**
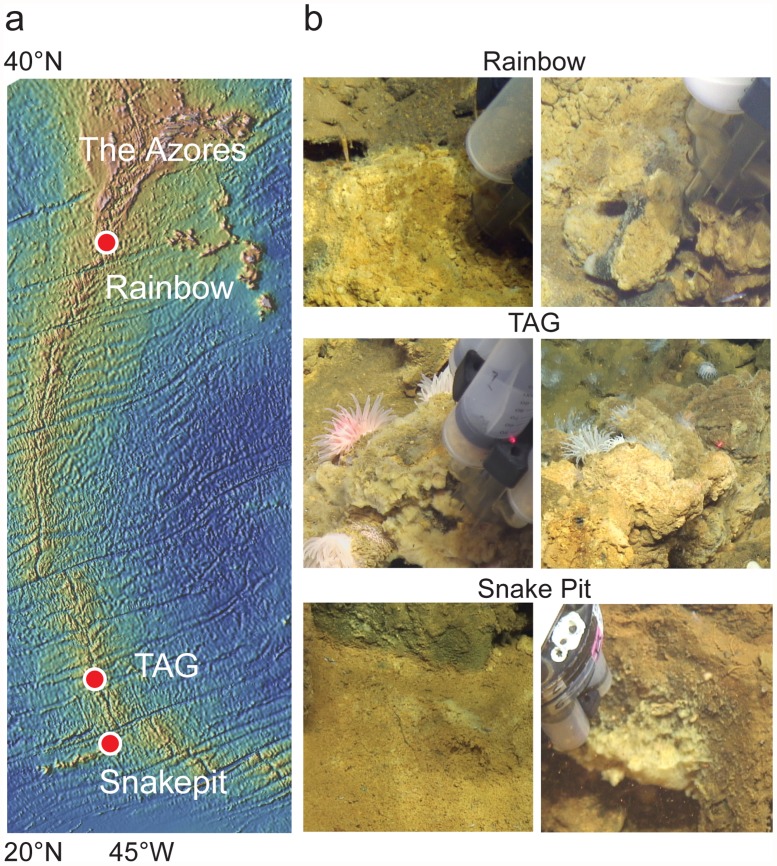
Study overview. **a)** Map showing the relative location of the Rainbow, TAG, and Snake Pit vent sites visited and **b)** representative images of mat material collected from Rainbow (top, RB-4A5 and RB-4A6), TAG (middle, TAG-5B1 and TAG-9B1), and Snake Pit (bottom, SP-7B2/7B4 and SP-7B5). Map was generated using GeoMapApp (http://www.geomapapp.org/, last accessed 8.4.2014). Photos taken by the ROV *Jason II* (courtesy of Woods Hole Oceanographic Institute).

Sampling of iron mats was conducted from October to November 2012, as part of the *Searching for NAnoparticulate Pyrite at the Mid Ocean RidgE* (SNAPMORE) expedition [43] aboard the R/V *Knorr* (cruise No. KN209–02). Samples were collected from three different off-axis vent sites along the Mid-Atlantic Ridge at Rainbow (dive J2664, 36°13′ 45.0431″N, 033°54′ 13.6052″W, ~2300 m), Trans-Atlantic Geotraverse (TAG) (dives J2665 and J2669, 26°9′ 45.9″N, 044°46′ 38.5″W, ~3600 m), and Snake Pit (dive J2667, 23°22′ 9.7″N, 044°57′ 8.3″W, ~3500 m) (Fig. 1a). Analysis of hot vent fluids showed significant differences between the fluids at these three sites [43]. The DSV *Jason II*, equipped with a newly developed precision microbial mat sampler [44], was used for discovery and collection of iron mat samples. In total we collected 50 discrete mat samples across the three sites. Roughly 20 mL was collected per sample, and replicate samples from the same exact site were combined into one sample. Aliquots of the combined samples were taken for structural analysis, single-cell sorting, and pyrosequencing. Samples for structural analysis (500 μL) were fixed in glutaraldehyde (2.5% final concentration) and stored shipboard at 4°C. Replicate 500 μL mat sample aliquots were reserved for single-cell sorting, stored at -80°C in a 1:1 ratio of glyASW (20% glycerol and 80% artificial seawater) and transported on dry ice. Remaining material was reserved for DNA analysis at -80°C and transported to the lab on dry ice. A total of eight samples were chosen for microbial community analysis (Table 1).

**Table 1:** Overview of sample details including site characteristics, pyrotag analysis, and cell counts.

Sample	Dist (m)	T_max_ (°C)	mbsl	Cell counts (mL^-1^)	N_seqs_	S_obs_	1/D*	Q*	d*	C*
RB-4A5	20.6	13.7	2295	2.4 x 10^7^ (2.3 x 10^6^)	33,523	325	5.16–5.33	99	.372	.996
RB-4A6	20.6	13.7	2295	2.0 x 10^7^ (3.1 x 10^6^)	4308	96	1.28–1.34	31	.872	.991
TAG-9B1	18.4	ND	3633	4.9 x 10^6^ (1.2 x 10^6^)	9958	345	5.47–5.86	111	.374	.987
TAG-5B1	14.8	5.1	3626	9.1 x 10^6^ (4.9 x 10^5^)	2506	202	3.44–3.97	79	.513	.967
TAG-5B2	17.2	5.1	3626	4.3 x 10^7^ (2.3 x 10^6^)	1032	89	5.86–7.24	30	.325	.965
SP-7B2	18.2	26.0	3505	4.6 x 10^7^ (3.0 x 10^6^)	2902	267	8.35–9.43	101	.246	.957
SP-7B4	21.0	26.1	3505	5.4 x 10^7^ (1.3 x 10^7^)	3763	502	2.76–3.07	275	.585	.928
SP-7B5	15.2	21.3	3505	3.0 x 10^6^ (2.9 x 10^5^)	2510	78	1.76–1.90	44	.708	.982

Site abbreviations; RB, Rainbow; TAG, Trans-Atlantic Geotraverse; SP, Snake Pit. Dist, distance (in meters) to closest sulfide chimney. T_max_, maximum temperature of diffuse flow associated with sample. mbsl, meters below sea level. Cell count reported as mean (SD). N_seqs_, total number of pyrotag reads. S_obs_, total number of OTUs.

*Alpha-diversity estimates based on sub-sampled pyrotag reads. *1/D*, Inverse Simpson Index; *Q*, Q-statistic; *d*, Berger-Parker Index; *C*, Good’s Coverage.

### Microscopy

To quantify the iron-oxyhydroxide morphotypes found in each sample, glutaraldehyde-preserved samples were mixed and then diluted to a concentration that allowed for microscopic visualization of the individual iron oxides with minimal clumping. Photomicrographs of 15 arbitrary microscope fields were taken at 40X magnification with bright-field illumination. Using the *ImageJ* image processing and analysis software package [45], iron-oxyhydroxides were manually traced and categorized as one of the following morphotypes: amorphous oxides (particles with no discernable shape); sheaths (long, hollow tubes, 1 to 2 μm in diameter); stalks (distinct helical twisted shape similar to those formed by strain PV-1 [46]); Y-guys (tubular oxides with at least one branching point); filaments (long, thin structures that could not be classified as a sheath, stalk or y-guy, either because these structures were highly mineralized—and thus undistinguishable—or was a previously unknown structure). *ImageJ* then measured the area of each traced structure, allowing for a comparison of the relative abundance of iron-oxyhydroxide morphotypes within each sample. An estimate of iron-oxyhydroxide material was calculated using the dilution factor and subsequently compared between samples. In conjunction with morphological analysis, direct cell counts (using epifluorescence microscopy of diluted mat material) were performed on triplicate counting slides as described previously [31]. Phase contrast and fluorescence microscopy were performed using an epifluorescence Olympus BX60 microscope equipped with a QICAM Fast CTD camera (Qimaging, Surrey, BC, Canada) as described previously [31].

### Sequence-based community analysis

**DNA extraction and 16S rRNA 454 pyrosequencing**. For this study we chose eight samples (two from Rainbow; three from both TAG and Snake Pit) for pyrosequencing analysis. Approximately 250 mg (wet weight) of mat material was extracted from each sample using a MoBio Power Soil DNA Extraction Kit (MoBio Laboratories), modified to include an initial phenol:chloroform:isoamyl alcohol (PCI) step. Briefly, 200 μL of bead solution was removed from each bead tube and replaced with 200 μL of 25:24:1 PCI (Sigma Aldrich). Samples were then extracted using the manufacture recommended protocol. Extracted DNA was sent to Research and Testing Laboratory (Lubbock, TX, USA) for pyrosequencing targeting the V4 hypervariable region (*E*.*coli* positions 531–997) using 530F (5′-GTG CCA GCM GCN GCG G-3′) and 1100R (5′-GGG TTN CGN TCG TTG-3′) [47]. All pyrosequencing libraries were deposited at the European Nucleotide Archive under the sample accessions numbers ERS527225–ERS527232, study accession number PRJEB6986.

**Sequence processing**. All sequence processing was performed using mothur v.1.33.0 [48] following previously published methodology [49] (mothur.org/wiki/Schloss_SOP, last accessed 4.11.2014). First, primers and barcodes were removed from each pyrotag sample, followed by a series of steps to translate flowgrams and reduce sequencing error. The dataset was then screened to remove reads that were less than 300bp, contained more than six homopolymers, and/or contained any ambiguities. Alignments were generated against a silva-based reference alignment (mothur.org/wiki/Silva_reference_files; last accessed 3.11.2014; 50,000 columns; 14,956 Bacterial, 2297 Archaeal, and 1238 Eukaryotic sequences) using the Needleman alignment method (k-tuple size, 8; match reward, +1; mismatch penalty, -3; gap extension penalty, -1; gap opening penalty, -5; search, kmer) [50,51]. Alignments were checked against a silva-based secondary structure-mapping file and then filtered to eliminate empty columns (trump, ‘.’; vertical, T; soft, 50). To reduce overestimation of richness due to pyrosequencing error [52], the dataset was also preclustered (*pre*.*cluster*) [53]. Putative chimeras were eliminated from the filtered alignments using uchime as implemented in mothur (template, self; settings, default) [54]. Finally, we constructed a phylip-formatted distance matrix (calc, one gap; count end penalty, true) [51], which was clustered using the average-neighbor algorithm [55].

**Taxonomic classification**. For taxonomic classification of pyrotag reads, we used a mothur-modified Greengenes reference taxonomy containing roughly 202,000 bacterial and archaeal sequences (last accessed 03.11.2014) [56] under the following parameters: search parameter, kmer; search method, bayesian; ktuple size, 8; match reward, +1; mismatch penalty, -1; gap open penalty, -2; gap extension penalty, -1; bootstrap cutoff, 80 [57]; iterations, 1000. Based on the resultant taxonomic summaries, we also calculated the consensus taxonomy for individual OTUs at 97% sequence identity [55].

**Estimates of community diversity**. *Beta* diversity was estimated using the *Bray-Curtis* similarity coefficient [58] against a random subsample of reads from each sample (based on the number of reads in the smallest sample, Table 1). The resultant similarity matrix was then clustered using the upgma algorithm [59] to create a *newick*-formatted dendrogram, visualized in FigTree v.1.4.0 (http://tree.bio.ed.ac.uk/software/figtree/, last accessed 07.22.2014). We estimated *alpha* diversity for each sample (based on the number of reads in the smallest sample, Table 1) using the following indices: the inverse *Simpson* index (1/*D*, non-parametric index, sensitive to abundant OTUs) [60]; *Berger-Parker* Index (*d*, proportional abundance of dominant OTU) [61]; the *Q*-statistic (*Q*, parametric index not skewed by very rare/abundant OTUs) [62]; and *Good’s coverage* (*C*, an estimate of sample coverage based on proportion of OTUs to reads) [63].

### Single-cell sorting and 16S rRNA amplification from SAGs

Two mat samples (RB-4A6 and TAG-9B1) were selected for single-cell sorting and 16S rRNA amplification. Prior to single-cell sorting samples were disrupted mechanically disrupted and diluted 1:10 in filter-sterilized artificial seawater. The complete process of single cell sorting and amplification has been described previously [64,65]. Samples were stained with SYTO-9 (Invitrogen) and sorted into 384-well plates containing 0.6 μL per well of TE buffer using a MoFloTM (Beckman Coulter) flow cytometer equipped with a CyCloneTM robotic arm. Cells were lysed and the DNA was denatured using cold KOH [66]. Genomic DNA was amplified using multiple displacement amplification (MDA) [67] in 10 μL final volume run at 30°C for 12–16 h, and then inactivated by 15 min incubation at 65°C. Of the 384 wells, 315 were dedicated for single cells, 66 were used as negative controls (no droplet deposition) and 3 received 10 cells each (positive controls). MDA products were diluted 50-fold in sterile TE buffer and 0.5 μL aliquots of the dilute MDA products served as templates in 5 μL real-time PCR screens targeting bacterial SSU rRNA genes using primers 27F and 907R. Forward (5-GTA AAA CGA CGG CCA GT-3′) or reverse (5′-CAG GAA ACA GCT ATG ACC-3′) M13 sequencing primer was appended to the 5′ end of each PCR primer to aid direct sequencing of the PCR products. All PCRs were performed using the LightCycler 480 SYBR Green I Master mix (Roche) in a LightCycler 480 II real time thermal cycler (Roche). 20 μL PCR reactions were set up for the PCR-positive SAGs and the 16S rRNA amplicons were sequenced from both ends using M13 targets. OTU-based analysis and taxonomic assessment were conducted using the approach described above. OTU-based analysis and taxonomic assessment were conducted using the approach described above. For comparative purposes we also included publically available SAGs from the Lō’ihi Seamount [68]. All SAGs from the MAR were deposited at the European Nucleotide Archive under the following accession numbers: LN589472–LN589572 (RB-4A6) and LN589573–LN589646 (TAG-9B1).

### Distribution of Zetaproteobacteria

A meta-analysis using publically available data was conducted to assess the distribution of Zetaproteobacteria across a range of comparable environments, including hydrothermal systems, non-marine iron-rich systems, marine sediments and seeps, and pelagic systems. In order to screen for signs of Zetaproteobacteria in other systems, raw pyrosequencing data (sff format) was downloaded from the Sequence Read Archive (SRA; http://www.ncbi.nlm.nih.gov/sra, last accessed 7.22.2014), the Visualization and Analysis of Microbial Population Structures (VAMPS; http://vamps.mbl.edu/, last accessed 7.22.2014), and metagenomics RAST server [69] (MG-RAST; http://metagenomics.anl.gov/, last accessed 7.22.2014) and analyzed as above. Because different variable regions were targeted across these studies, it was not possible to look for shared OTUs, and as such we focused exclusively on taxonomic content. Any reads returning a taxonomic classification of ‘Unclassified Proteobacteria’ (using the above methodology) were further scrutinized using the SINA alignment and classification service under default settings (http://www.arb-silva.de/aligner/, last accessed 7.22.2014) [70]. This allowed us to ensure that any putative Zetaproteobacteria reads were not missed during the initial screen. Positive Zetaproteobacteria hits were also screened through the SINA service to identify any potential false positives. It is important to note that we have independently analyzed thousands of publicly available 454 and Illumina samples from numerous environments (including phyllosphere, compost, soil, insects, marine mammals and terrestrial mammals, freshwater, etc.) and found no evidence of Zetaproteobacteria. Thus, we chose to focus this meta-analysis on environments where Zetaproteobacteria were likely to be detected

## Results

At all three vent sites (Rainbow, TAG, and Snake Pit) it was possible to find prototypical iron mats associated with diffuse hydrothermal venting proximal to primary black smoker fields. Examples of iron mats from the three sites are shown in Fig. 1b. In all cases, iron mats were highly localized around discrete vent orifices and did not accumulate into expansive microbial mat ecosystems similar to those observed at sites like the Lō’ihi Seamount [31]. While there was variability in the morphology of individual mats, typical mats were mound formations, 5–10 cm wide and 10–20 cm high, that often occurred in clusters with several individual mats within a few square meters. The total volume of individual iron mats was small, and due to weather conditions and technical issues, it was not possible to do surveying or more extensive geochemical analysis of the sites. Thus we do not have estimates of overall prevalence of iron mats at any of the three sites, or a detailed geochemical analysis of the fluids. The temperature of vent fluids associated with iron mat accumulations ranged from near ambient (~2°C) up to 26°C (Table 1). Cyclic voltammetry was used to measure iron(II) and oxygen concentrations at vents associated with TAG and Snake Pit before and after mat collection. Immediately above mat surfaces, dissolved oxygen ranged from 78–152 μM, iron(II) averaged 25 μM, and sulfide was not detectable (0.2 μM detection limit) [71].

### Microbial diversity of MAR iron mats

Approximately 90,000 16S rRNA pyrotag reads were generated from the three MAR sites. After sequence processing and quality control, the dataset contained 60,689 high-quality reads (~470bp average length). Library sizes ranged from 1032 to 33,532 reads (average, 7563) (Table 1). Distance matrix clustering resulted in 1264 total OTUs at 97% OTU identity and 450 total OTUs after removal of singleton and doubleton reads.

**Fig 2:**
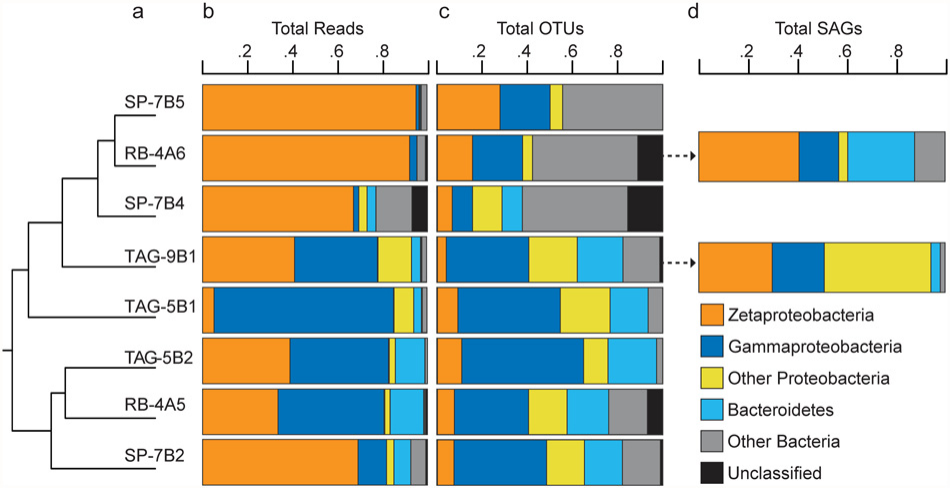
Broad-scale composition of iron-oxidizing microbial communities at the Mid-Atlantic Ridge. **a)** OTU-based estimate of community similarity based on a random subsample of reads from each sample. Samples RB-4A5 and RB-4A6 were collected from Rainbow, TAG-9B1, TAG-5B1, and TAG-5B2 from TAG, and samples SP-7B2, SP-7B4, and 7SP-B5 from Snake Pit. **b**) Relative abundance (% total) of pyrotag reads from major bacterial groups. **c**) Relative abundance of OTUs (with singletons/doubletons removed) assessed at 97% OTU identity. **d**) Relative abundance of SAGs from two samples (RB-4A6 and TAG-9B1) based on single-cell sorts of mat material. Color scheme for **b**–**d** adapted from the Color Universal Design (http://jfly.iam.u-tokyo.ac.jp/color/, last accessed 8.8.2014).

Whole community beta diversity estimates (OTU-based) of samples from Rainbow, TAG, and Snake Pit indicated that there was as much within-site variability as there was between-site variability (Fig. 2a). Taxonomic analysis of MAR iron mats (across all samples) revealed representatives from over 30 bacterial phyla; however only 5 phyla comprised greater than 1 percent of total reads (Fig. 2b). Roughly 45 percent of total pyrotag reads corresponded to the class Zetaproteobacteria (mean across samples [SD], 55.2% [31.1]; range, 5.11–95.0%) while Gammaproteobacteria (28.4% [28.3]; range, 1.27–79.9%) and Bacteroidetes (5.94% [5.51]; range, 0.492–14.9%) were also well represented in several, but not all, samples (Fig. 2b). In contrast, Zetaproteobacteria contributed much less to total OTU richness (mean [SD], 8.95% [1.67]; range, 3.69–4.6%) than either Gammaproteobacteria (23.2% [4.19]; range, 6.79–40.2%) or Bacteroidetes (13.9% [1.70]; range, 6.25–19.4%) (Fig. 2c).

We also recovered 101 high quality SAGs from RB-4A6 and 74 from TAG-9B1 spanning the V1–V4 regions of the 16s rRNA gene (average length, 926 base pairs). In general, taxonomic content of the SAG datasets was similar to the overall patterns observed in the corresponding pyrotag datasets; specifically, SAGs were also dominated by Zetaproteobacteria, Gammaproteobacteria, and Bacteroidetes (Fig. 2d). That said, roughly one-third of the SAGs from TAG-9B1 were identified as Epsilonproteobacteria (in contrast to 14% of total reads from TAG-9B1 pyrotag dataset) and 15% of the SAGs from RB-4A6 were identified as Bacteroidetes (in contrast to less than 1% total reads from RB-4A6 pyrotag dataset) (Fig. 2d).

**Fig 3:**
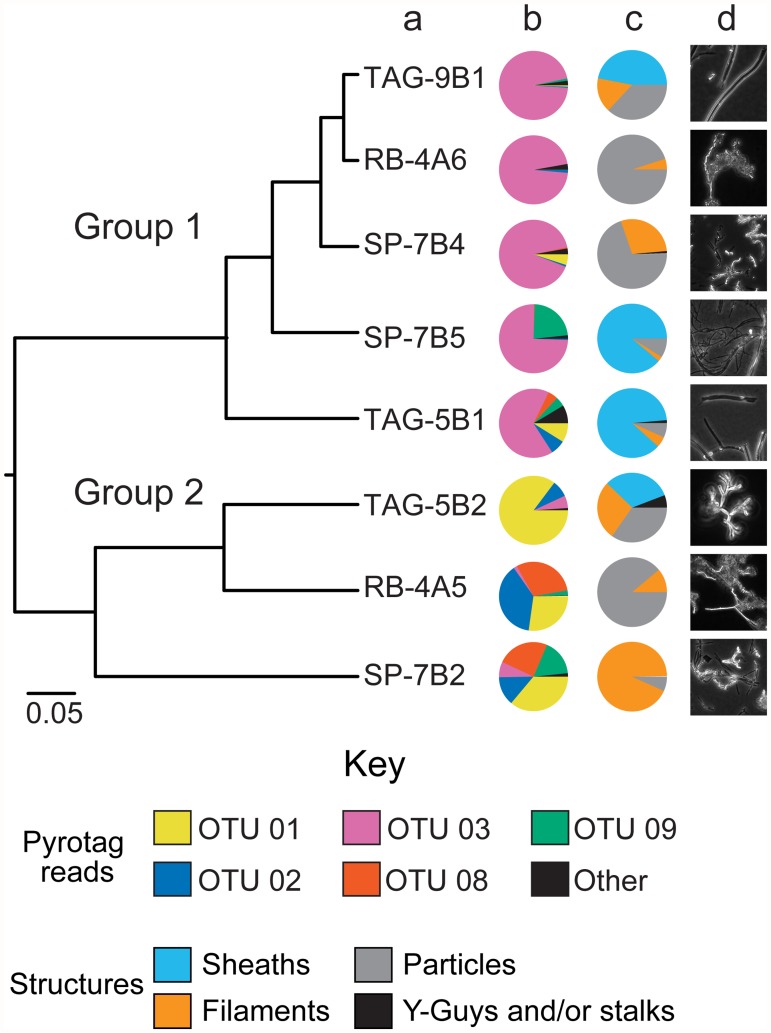
Zetaproteobacteria diversity. **a**) Bray-cutis similarity estimates of community composition based solely on reads from Zetaproteobacteria. Datasets were subsampled to account for uneven sampling. Pie charts indicating **b**) the relative abundance of dominant Zetaproteobacteria OTUs, and **c**) the relative abundance of structural types from each sample. **d**) Light microscopy images of iron oxyhydroxide structures observed in the different mat samples. Color scheme for **b** and **c** adapted as in Fig. 2.

Alpha diversity estimates using both the inverse *Simpson’s* index (*1/D*) and *Q*-statistic (*Q*) indicated that, in general, mat samples at the MAR contained low bacterial diversity (Table 1) compared to other marine environments (Table 2). *Berger-Parker* (*d*) analysis showed that the proportional abundance of dominant OTUs was high, meaning that samples were generally dominated by a single OTU (Table 1). In all samples the dominant OTU was a member of the Zetaproteobacteria, however it was not the same OTU across all samples (Fig. 3). Samples in Group 1 (Fig. 3a) were dominated by OTU 03 (Fig. 3b), while Group 2 samples contained greater Zetaproteobacteria OTU richness, including OTUs 01, 02, and 08.

**Table 2:** Meta-analysis of publically available 16S pyrosequencing datasets.

Environment	N	n_seqs_	S_obs_	n_seqs_ Zeta	1/D*	Q*	d*	Ref
***Non-marine Fe-rich systems***								
Acid mine drainage (SE China) [V4]	59	125,706	1531	0	4.32	619	0.453	[90]
Freshwater iron seeps [V4]	28	101,921	9932	0	42.5	4189	0.121	[91]
***Hydrothermal systems-fluids***								
Azores [V6]	15	229,985	2748	431	50.4	899	0.087	[86]
Juan de Fuca Ridge [V13]	6	27,878	2071	0	72.6	627	0.053	[92]
MAR (Logatchev) [V13]	46	237,388	2037	4	11.6	823	0.244	[93]
Seamounts (Mariana Arc, Lō’ihi) [V6]	41	319,074	6448	1682	25.2	1821	0.110	[87]
***Hydrothermal systems-vent deposits***								
E. Lau Spreading Center [V4]	36	324,951	3432	264	28.9	439	0.113	[94]
Lau (sulfides) [V6]	10	83,243	1817	1607	18.0	518	0.185	†
Lost City (carbonate chimney) [V6]	4	29,951	590	0	10.4	171	0.253	[95]
E. Pacific Rise (inactive sulfides) [V6]	15	188,238	2464	2724	31.8	934	0.107	[85]
Guaymas Basin [V4]	13	102,760	1055	0	23.0	297	0.168	†
MAR (Rainbow, TAG, Lucky Strike) [V4]	16	132,302	1006	80	23.1	162	0.110	[96]
***Sediments***								
Cariaco Basin [V6]	13	62,401	1837	0	14.3	594	0.187	[86]
Levantine continental margin [V13]	1	1083	104	735	18.0	203	0.157	[5]
Pacific Basin [V6]	8	138,549	1388	0	31.7	413	0.073	[86]
S. Pacific Gyre [V6]	8	117,140	1990	4	31.4	606	0.175	[97]
Station M [V6]	16	227,413	5702	2	127	2051	0.049	[86]
Various (Deep subseafloor) [V6]	7	106,348	5671	85	25.2	2356	0.179	[86]
Guaymas Basin (methane seeps) [V6]	10	89,717	5075	1	105	1773	0.045	[86]
***Water column***								
Azores surface waters [V6]	13	233,150	2231	0	35.3	714	0.116	[86]
Baltic Sea transect [V4]	208	242,903	2488	6	90.7	726	0.055	[98]
E. Atlantic [V6]	16	244,734	2080	3	21.8	678	0.186	[99]
N. Atlantic [V6]	47	431,625	1943	8	6.54	688	0.373	[4]
**Iron mats (MAR) [V4] This study**	**8**	**60,684**	**1264**	**26,692**	**9.18**	**491**	**0.211**	

Datasets obtained from the SRA, VAMPS, and MG-RAST. The 16S rRNA hypervariable region assessed in each study is noted in brackets. Raw data was processed as described in the Materials and Methods section and each dataset screened for the presence of Zetaproteobacteria. N indicates the number of samples; n_seqs_ the total number of reads; S_obs_ the total number of OTUs.

*Alpha-diversity estimates based on sub-sampled pyrotag reads. *1/D*, Inverse Simpson Index; *Q*, Q-statistic; *d*, Berger-Parker Index.

†Unpublished datasets used with permission from A.-L. Reysenbach.

Of the 450 total OTUs found in this study (97% OTU identity), only OTUs 02, 03, and 56 (all Zetaproteobacteria) were found in all eight samples, accounting for roughly 28 percent of total reads (range, 4.30–91.4% per sample). In fact, only 12 other OTUs were found in five or more samples, accounting for 62.8 percent of total reads (range, 50.2–97.9%). Of these, 6 OTUs were Gammaproteobacteria (Methylococcales, Thiotrichales, and ‘Unclassified’), 2 Bacteroidetes (Flavobacteriales), 1 Epsilonproteobacteria (Campylobacterales), as well as 3 additional Zetaproteobacteria OTUs.

### Structural analysis and cell counts

All samples analyzed in this study contained particle and filament-like structures, though the abundance of both varied considerably across samples (Fig. 3c). Although sheaths were only detected in four of the eight samples, they accounted for a high percentage of total structural area in those samples. Only one sample (TAG-5B1) showed any clear evidence for stalk-like structures, characteristic of species like *Mariprofundus ferrooxydans*; however, even in this sample stalks accounted for just 1.3 percent of total area. Similarly, Y-structures were only found in two samples (TAG-5B1 and SP-7B4), again representing a small percentage of total area. There was no apparent relationship between structural content and Zetaproteobacteria OTUs. For example, structure analysis indicated that both RB-4A5 and RB-4A6 (Rainbow samples) were composed almost entirely of filaments and particles (Fig. 3c) yet had very different Zetaproteobacteria OTU profiles as well as overall community composition. That said three of the four samples with an abundance of sheaths also contained a high proportion of pyrotag reads corresponding to OTU 03.

## Discussion

This study confirms the discovery of iron-oxidizing microbial mat communities associated with diffuse flow venting at the Mid-Atlantic Ridge (MAR). We used pyrosequencing along with microscopy and single-cell sorting to demonstrate the presence and relative abundance of iron-oxidizing Zetaproteobacteria at the Rainbow, Trans-Atlantic Geotraverse (TAG), and Snake Pit hydrothermal vent fields along the MAR. Light microscopy revealed iron-oxyhydroxide structures consistent with biogenic oxides produced by other neutrophilic FeOB including iron encrusted sheaths, filamentous helical stalks, and Y-shaped filaments [35].

### Diversity of MAR iron mats

In addition to its dominant presence in pyrotag libraries, Zetaproteobacteria was the only taxa with OTU representatives shared across all samples. This finding further supports the hypothesis that the presence of iron(II) is an important driver of Zetaproteobacteria population structure. Given that we found a limited number of other taxonomic groups shared across a majority of samples, it remains unclear the extent to which such taxa are restricted to iron mats and what role(s) they may play in community-level processes and iron cycling. Our finding that samples from the same vent field contained different diversity of Zetaproteobacteria OTUs suggests that local physiochemical conditions (e.g., fluid temperature and flow, nutrient availability, iron and oxygen concentration)—conditions that likely shift at the centimeter to meter scale (as was found at the East Pacific Rise for macro-faunal assemblages [72])—could play a major role in structuring Zetaproteobacteria diversity. Furthermore, whole community clustering analyses indicated that overall community similarity was not entirely related to geographic location, suggesting that local physiochemical conditions could also play a substantial role in governing general bacterial diversity within these mats as well. Fine-scale transect-based sampling coupled with co-registered biogeochemical analysis could help reveal the underlying factors driving both community structuring and Zetaproteobacteria diversity within these systems. It remains to be seen whether iron mats contain core community members beyond the Zetaproteobacteria. Future studies should investigate the surrounding water column and sediments to determine the degree of habitat restriction at iron seeps and further understand the influence of iron seeps on community structure in the vicinity of iron mats.

### Shrimp associated Zetaproteobacteria at the MAR

Though this is the first report of iron mats and free-living Zetaproteobacteria at the MAR researchers investigating the abundant swarms of *Rimicaris* shrimp at the Rainbow vent system noted that many shrimp had iron-oxyhydroxides associated with their carapaces and presented microscopic evidence for an association between putatively symbiotic bacterial cells and iron-oxyhydroxides [73]. In addition, a recent community metagenomic analysis reported Zetaproteobacteria associated with the gill chamber of the shrimp, *Rimicaris exoculata*, from the Rainbow hydrothermal system [74]. As these researchers noted however, Zetaproteobacteria comprised a small fraction of the total metagenome, which was dominated by sulfur-oxidizing bacteria belonging to the Epsilon- and Gammaproteobacteria. We retrieved three unique, partial 16S rRNA Zetaproteobacteria reads from the *Rimicaris* metagenome with enough sequence overlap to compare with the single amplified genome (SAG) samples from Rainbow and TAG. Comparative sequence analysis indicated that five SAGs from the Rainbow sample were more than 99 percent identical to one of the *Rimicaris* reads (97% identical to the other two *Rimicaris* reads). Interestingly, these five SAGs were not found in the SAG libraries from either TAG (this study) or the Lō’ihi Seamount [68]. Furthermore, the SAGs from both TAG and Lō’ihi were no more than 95 percent identical to any *Rimicaris* sequences (most were only 90–92% identical). Given the limited sample size and truncated region of 16S rRNA analyzed, it is difficult to draw any conclusions; however, these findings suggest that local iron mats could be a potential source of Zetaproteobacteria associated with the gill chamber of *Rimicaris* and that gill-associated Zetaproteobacteria OTUs may be geographically restricted. Coordinated microbial community analyses of iron mats and *Rimicaris* populations across the MAR could reveal whether shrimp associated Zetaproteobacteria are in fact endemic to specific vent systems. During our expedition it was common to observe shrimp moving across the surface of iron mats at all three sites; however shrimp abundance was far lower than the dense swarms associated with the black smokers [75]. Though we observed shrimp associated with mat surfaces, we were unable to determine if the shrimp were actually grazing on iron mats. Durand et. al. [76] reported the presence of iron-oxides particles in the guts of *Rimicaris* from Rainbow and TAG however molecular analysis did not indicate the presence of Zetaproteobacteria. Whether grazing on iron mats could be a possible route for colonization of shrimp by FeOB remains to be determined.

### Biogeochemical implications

The three sites along the MAR exhibit different geological settings. Both TAG and Snake Pit are associated with ultramafic rocks while Rainbow is a basalt-hosted system [77]. The data presented here do not indicate that the basal geological differences between the three sites play a major role in determining microbial community structure; we did not find evidence that specific community assemblages were associated with either basalt versus ultramafic rock types. This is consistent with a recent study that did not find strong correlations between populations of hydrogen- and sulfur-oxidizing bacteria at either basalt or ultramafic-hosted systems at vents located on the southern MAR [78]. Thus it would appear that local factors (e.g., temperature, redox conditions, iron(II) concentration, and/or fluid flow rate) might be more important drivers of community structure in iron mat ecosystems rather than geological setting.

It is also interesting that there are so few reports of FeOB associated with the MAR or other vent habitats at crustal ridge systems. Despite numerous expeditions to the various vent sites on the MAR, there is only one previous report suggesting the presence of free-living FeOB. In 1990, Wirsen et. al., [79] collected samples of reddish-brown flocculent material from a site at TAG using the DSV *Alvin* and reported the presence of sheath-like structures. At the time, these were thought to be evidence for a *Leptothrix* or *Sphaerotilus*-like sheath forming iron-oxidizers. This was before the discovery that marine FeOB belong to the Zetaproteobacteria and are unrelated to freshwater FeOB like *Leptothrix* and *Sphaerotilus*, both of which are Betaproteobacteria [80]. Nonetheless, our discovery of sheathed FeOB at TAG coupled with the observations by Wirsen et. al. [79] suggests that iron-oxidizing communities are likely consistent features of the TAG vent field ecosystem.

At present, we have little appreciation for how common or widespread microbial iron mats may be, either at the MAR or other crustal spreading centers. Additionally, it is uncertain whether iron mats are only found associated with easily detected high-temperature vent fields. In isolation, low-temperature, diffuse flow iron mat ecosystems are more difficult to locate due to minimal temperature anomalies and less active plumes [32]. A recent transect across the South Atlantic Ocean investigating dissolved iron levels throughout the water column, reported higher than expected iron concentrations in the region of the MAR [81]. The authors suggested that hydrothermal iron inputs associated with crustal ridge spreading centers was the most likely source; however the specific sources of iron, either diffuse flows or black smoker systems have yet to be identified. FeOB could be influencing the flux of iron into the ocean through a complex set of interactions, including the production of poorly crystalline hydrous ferric oxides that sequester iron oxides in microbial mats. At the same time, FeOB can release a fraction of these micro- and nanoparticulate oxides into the water column [43,82,83]. Until we know more about the extent of iron sources at hydrothermally active regions it is possible that iron(II)-based chemosynthetic environments are being underestimated in the deep ocean; however until we know more about the ubiquity of Zetaproteobacteria and associated diffuse flow iron(II)-rich hydrothermalism, it is difficult to assess their role in controlling the iron flux in the deep ocean.

### Meta-analysis of Zetaproteobacteria distribution

The results described here contribute to a growing number of studies reporting the presence of Zetaproteobacteria in marine ecosystems where iron(II) is present at micromolar concentrations, as opposed to the low nanomolar concentrations typical of seawater. A meta-analysis of 16S rRNA clone libraries across a range of dark ocean habitats conducted by Orcutt et. al., [84] found that Zetaproteobacteria did not occur at high frequencies and appeared to be restricted to hydrothermal and basalt deposits. To further assess the extent of Zetaproteobacteria distribution across marine systems, we conducted a meta-analysis of 23 publically available 16S rRNA pyrosequencing datasets encompassing a variety of marine environments and non-marine iron-rich systems (Table 2). This analysis of over 3.8 million 16S rRNA pyrotag reads from nearly 650 samples revealed remarkably little evidence of Zetaproteobacteria in marine environments outside of iron-rich systems (Table 2). Not surprisingly, we found no evidence for Zetaproteobacteria in either freshwater iron seeps or acid mine drainage (Table 2). An appreciable number of Zetaproteobacteria (> 0.1% total reads) were, however, detected in four datasets sets: East Pacific Rise inactive sulfides [85]; Lau Basin active sulfide deposits [86]; Azorean shallow vents [86]; and seamount fluids [87] (Table 2). In the first three instances, the majority of Zetaproteobacteria reads originated from a single sample within each dataset (7M24 [82%], ALR_0005_2005_04_22 [98%], ASV_0015_2001_08_24 [99%], respectively). In the seamount fluids dataset, 90 percent of the Zetaproteobacteria reads were from three samples (LOIHI-PP1, LOIHI-PP2, LOIHI-PP5). Based on our interpretation of associated metadata, all samples containing Zetaproteobacteria were suggestive of FeOB habitats. The East Pacific Rise sample containing Zetaproteobacteria was collected from iron-rich massive sulfides and the Lau sample collected from a microbial mat (the only mat sample in that dataset). The Azorean shallow vent sample was collected from water around a “red biofilm” and the three seamount fluid samples were collected from the summit of Pele’s Pit (Lō’ihi Seamount), a site known to contain a preponderance of Zetaproteobacteria [31,32].

It is also important to point out that marine iron(II)-rich ecosystems are not exclusively associated with hydrothermal activity. The Levantine basin sample shown in Table 2 was collected from continental shelf sediments, at a water depth of 800 meters, where iron, iron-oxyhydroxide stalks, and Zetaproteobacteria were detected in high concentrations associated with localized bioturbation of soft sediments [5]. Furthermore, studies from Maine [88] and China [89] have shown that iron-oxidizing Zetaproteobacteria can rapidly colonize steel surfaces placed in coastal environments indicating that there must be local reservoirs of FeOB, perhaps originating from more iron-rich sediments.

Another intriguing aspect of this meta-analysis is the general paucity of Zetaproteobacteria in samples collected from the same (Rainbow, TAG) or similar (Lucky Strike) hydrothermal systems on the MAR (Table 2). Based on GPS coordinates, iron mat samples from Rainbow and TAG were collected within 80 and 40 meters of the corresponding publically available datasets, respectively (Table 2, see MAR datasets under Hydrothermal systems-vent deposits). This shows that at a local scale Zetaproteobacteria may dominate in microaerobic zones where iron(II) is abundant compared to background seawater; but just a few meters away, where iron(II) concentrations may be low and another electron donor (e.g. reduced sulfur species) is more abundant, Zetaproteobacteria are barely detectable. Conversely, based on our data there is little evidence for sulfur-oxidizing bacteria in the microbial iron mats at the MAR as sulfide was not detectable in the mats studied. Thus, hydrothermal systems on the MAR appear to harbor at least two distinct and prevalent chemolithotrophic microbial systems.

## Conclusion

In this study we demonstrate the presence of iron-oxidizing Zetaproteobacteria at three well-studied vent sites on the MAR proximal to black smoker vent fields. Based on this and previous studies, Zetaproteobacteria appear to be restricted to iron-rich marine environments and are generally absent within other marine systems where iron is unlikely to be available at concentrations that can support lithotrophic growth. Continued investigations may uncover Zetaproteobacteria associated with non-iron systems and comparative genomic analysis may help explain why this group appears to be so highly specialized. Given that metabolically available iron(II) may be ubiquitous (if not patchy), Zetaproteobacteria likely contribute more to deep ocean primary production than is currently appreciated.
